# Association Between Psoriasis and Depression: A Traditional Review

**DOI:** 10.7759/cureus.9708

**Published:** 2020-08-13

**Authors:** Faryal Mustansir Sahi, Ayesha Masood, Nuaman A Danawar, Andrew Mekaiel, Bilal Haider Malik

**Affiliations:** 1 Dermatology, California Institute of Behavioural Neurosciences and Psychology, Fairfield, USA; 2 General Surgery, California Institute of Behavioural Neurosciences and Psychology, Fairfield, USA; 3 Internal Medicine, California Institute of Behavioural Neurosciences and Psychology, Fairfield, USA

**Keywords:** depression, suicidality, chronic inflammation of skin, low self-esteem, psoriasis pathophysiology

## Abstract

Psoriasis is a chronic inflammatory disease of the skin with multiple comorbidities, depression being one of them. Psoriasis affects the personal, social, and sexual lives of the patients resulting in psychological strain.

Psoriasis and depression amplify each other. Supporting evidence has proven multiple common mechanisms between the two diseases: inflammatory overlap, genetic evidence, low vitamin D3, and melatonin levels are common in both psoriasis and depression. Fear of social rejection and self-stigmatization act as a fuel to fire inflaming depression in psoriatic patients.

The study explains the link between psoriasis and depression and their effects on quality of life. There is a need to highlight the importance of addressing the psychological effects of psoriasis along with its physical aspects for better treatment outcomes.

## Introduction and background

The World Health Organization reported psoriasis as a severe global problem in 2016 [1]. Its prevalence ranges between 0.09% in the USA and 8.5% in Norway, affecting 1.3% of African Americans and 2.5% of Caucasians [2,3]. Psoriasis patients suffer from embarrassment due to visible physical symptoms. As a result, they have low self-esteem, anxiety, and become depressed [4,5]. According to a study report of 127 psoriasis patients, 9.7% of patients wished they were dead at the time of the study, while 5.5% had suicidal ideation [6].

Psoriasis is a chronic, inflammatory, immune-mediated disease of the epidermis with systemic involvement. Psoriatic lesions appear as itchy, reddish raised plaques covered with silvery scales. Generally considered to be a skin disease, psoriasis is associated with multiple comorbid disorders, including mood disorders. Psoriatic lesions can appear anywhere on the body, and the ones appearing on the uncovered areas of the body bring forth the feelings of being unattractive and frustration; patients tend to isolate themselves socially and start being alone, which makes them depressed [1]. Psoriasis affects the social, personal, and sexual lives of the patients, thus reducing the quality of life (QoL) and causing psychological strain. Lives at home, school, and workplace are affected [7-16]. Myths that psoriasis is a contagious disease stigmatize and exclude the patients from schools, workplaces, and swimming pools, devastating their social lives [1]. Studies have proven that the depressive symptoms were exacerbated in patients who felt stigmatized in social situations as compared to those who were not stigmatized by these situations [8].

Generally, the feelings of being physically unattractive result in low self-esteem and depression. This article will explain the relation between psoriasis and depression, their pathophysiologies, and effects on daily lives. We intend to find out if it is just the physical appearance of the condition that results in depression or are there other factors involved. Is everyone affected equally by the psychological effects of the disease or if there is a vulnerable group? Multiple comorbid conditions associated with psoriasis could play a role in development of low mood and depression.

Treatment of psychosocial morbidity is essential when assessing the severity of psoriasis as it plays a significant role in the patient's understanding of the disease and the disease course [7]. Understanding the relationship between depression and psoriasis would help in better management of the disease by the clinicians. The patients who are psychologically depressed are mostly non-compliant and focus on the negativity. They might think that they would never get rid of the disease despite the treatment. Managing the psychosocial part of the illness, along with its physical aspect, would give better treatment outcomes.

## Review

Inflammatory and immunological changes in psoriasis

Psoriasis is a chronic inflammatory disease of the skin and has a relapsing and remitting course. Studies on the pathophysiology of psoriasis have shown that psoriasis occurs as a result of inflammatory and immunological reactions. In psoriasis, macrophages, T-cells, and dendritic cells migrate towards the epidermis and away from the dermis, releasing the inflammatory cytokines like tumor necrosis factor-alpha (TNF-α), interleukin 1β (IL-1β), and IL-6, IL-22, or a type-1 cytokine profile (IL-2, interferon-γ [IFN-γ], and TNF-α) [9]. Defect in regulatory T-cells and the regulatory cytokine IL-10 is present in psoriasis. Keratinocytes are triggered by the chemical messengers from dendritic cells and T-cells to release cytokines, which cause more inflammation by signaling the downstream inflammatory cells [10]. Dendritic cells act as a bridge between the innate and the adaptive immune system and activate these systems in psoriatic lesions [11]. Brain-derived neuropathic factor (BDNF), which plays an essential role in neuropathic and mental disorder, was found to be associated with psoriasis [12]. These BDNF levels are decreased in depression and psoriasis [13].

The discussion so far supports that several inflammatory markers are involved in psoriasis and are crucial to the pathogenesis of the disease. The study by Palfreeman et al. focuses mainly on the management of psoriasis and psoriatic arthritis [9]. However, Nestle et al. in their study used mice as models for understanding the molecular basis, genetics, and treatment therapies of psoriasis [10]. The study explains in detail the pathogenesis of psoriasis and inflammatory markers that are crucial to the pathogenesis of the disease.

Immunological changes in major depression

Depression is a common mental health problem that causes patients to have low mood and loss of interest in daily activities. According to a study, high levels of proinflammatory cytokines (TNF-α and IL-6) are found in patients suffering from depression, usually in the absence of any inflammatory disease [14]. In contrast, chronic inflammation can cause various mood disorders, including depression [15]. Peripheral blood circulation and cerebrospinal fluid of depressed patients have shown an increased level of IL-1-beta and TNF-α [16]. Also, in a study on dogs, high inflammatory cytokines caused sickness behavior and depression; this may also happen in humans [17]. The therapeutic administration of cytokine INF-α further proved the role of inflammation in depression, and this caused depression in half of the patients [18]. According to another study, neurotransmitter metabolism, neuronal health, and neuronal activity are negatively affected by elevated inflammatory cytokines. These peripherally released cytokines reach the central nervous system via molecular, cellular, and neural pathways and enhance inflammation in mood-related brain regions [19].

Depression generally is known to be caused by disturbance in the levels of different neurotransmitters in the brain. Several inflammatory markers are seen to overlap between psoriasis and depression. Moon et al. considered the impact of psoriasis beyond the skin as unrecognized and aimed to explore the different mechanisms that link psoriasis with psychological distress [17]. Their study included a checklist that was completed by dermatologists and patients; data collected were used to find out how assessment of disease severity and health-related QoL differs in these two groups. Raison et al. in their study explained the role of inflammatory innate immune responses in depression and its treatment implications [18]. The study describes the inflammatory markers that play a substantial role in the pathogenesis of depression.

Common mechanisms in psoriasis and depression

Contrary to common belief, it is not just psoriasis that can lead to depression. Psoriasis can also be caused by major depression due to immunological and neurochemical phenomenon [20]. Depression, generally considered to be a mental health disease, can also lead to pathologies of the skin and other organs such as the heart [21]. The possibility of a relationship between psoriasis and major depression has been shown by many studies [13]. In two separate studies done by Gupta et al., an increase in psoriasis flare-ups and pruritus severity were found to correlate with an increase in stress and depressive symptoms [22,23]. Studies done by Akay et al. and Esposito et al. showed the correlation between psoriasis and depression [24,25]. Genetic evidence also suggests that there exists some relationship between psoriasis and depression. Genetic variations in the serotonergic system that includes a variable number of tandem repeat polymorphism intron 2 of the 5-HT gene and polymorphism of serotonin receptors seem to play a role in the pathogenesis of psoriasis and depressive symptoms in psoriasis patients [26].

Furthermore, vitamin D3 deficiency is involved in the pathogenesis of both psoriasis and depression [27]. Vitamin D3 metabolites have been shown to play a role in normal differentiation and growth of keratinocytes, and its supplementation has shown to improve the skin in some conditions, including psoriasis [28]. In psoriasis patients, low vitamin D3 concentration caused decreased counts of circulating T regulatory cells [29]. Thus, 25(OH)D3 has a role in the pathogenesis of psoriasis, it acts as an immunomodulator and prevents excessive Th1 and Th17 responses [27]. For this reason, skin-related diseases get benefit from vitamin D3-related analogs. Also, vitamin D3 deficiency causes behavioral disorders in animals, and its absolute and relative deficiency has shown to increase the risk of mood disorders in humans [30,31]. Vitamin D3 deficiency is also linked to increased concentrations of inflammatory markers, and these inflammatory markers are crucial to the pathogenesis of depression, as discussed earlier [30]. Vitamin D might be directly involved in the development of depression, as central nervous system contains vitamin D receptors [32]. These receptors are also present in structures such as the hippocampus and prefrontal cortex, which are involved in mood control [33]. Therefore, low serum concentrations of vitamin D3 seem to play a role in the pathogenesis of both psoriasis and depression, providing some evidence of how these two can co-exist. Melatonin levels have also shown to relate psoriasis with depression [13]. Although the major function of melatonin is to regulate the sleep cycle, studies have found its additional influence on the immune system along with other inflammatory markers that affects the levels of TNF-α, IL-6, and IL-8 [34]. Additionally, low levels of melatonin were found in the patients having skin diseases, including psoriasis, and normalization of the melatonin levels was observed with reduction of the depressive state and clearing of the psoriatic lesions [34,35].

Also, treating psoriasis improves depression. Depression in psoriasis, which is partially attributed to raised proinflammatory cytokines, can be reduced by using biologics [36]. Depressive symptoms and proinflammatory cytokines can be reduced by treating with pharmacological anti-inflammatory interventions [37].

In light of the pieces of evidence presented above, depression and psoriasis seem to have a bidirectional relationship. Depression can lead to psoriasis and psoriasis can cause depression. Genetic evidence, inflammatory overlap, low vitamin D3, and melatonin levels in both psoriasis and depression set up a connection between these two diseases. Therefore, depression in psoriasis is not merely because of physical appearance of the disease, but other factors are responsible for depressive symptoms in psoriasis. Tohid et al. carried a study to explain the inflammatory and immunological mechanisms taking place in psoriasis and depression and how they overlap between the two diseases [13]. Besides, their research includes genetic evidence to support the bidirectional relationship between psoriasis and depression. Pietrzak et al., in their study, described the mechanisms that are common between psoriasis and depression [27]. Their research includes dysfunction of the hypothalamic-pituitary-adrenal axis and vitamin D3 deficiency as similarities between the two diseases, in addition to the inflammatory markers that were explained in the study by Tohid et al.

Feelings of depression and stigmatization in psoriasis and its aspects by gender

Skin is the most visible organ of the body, especially the uncovered areas like head, neck, hands, and feet, and any lesions appearing in these areas might stigmatize the patients, especially when it is topped up by negative reactions from society [38,39]. Although psoriasis patients generally feel underconfident and ashamed, these feelings are worse when it comes to their sexual relationships [40]. Patients start to dislike their physical appearances and feel that others see them the way they see themselves [41]. Psoriasis patients are more prone to depression as compared to the general population [42]. Also, among dermatological diseases patients with psoriasis have more risk of developing depression than any other disease [43]. Patients' QoL is decreased by continuous exposure to stress, feelings of stigmatization, and disapproval of one's external appearance along with the chronic nature of the disease, contributing to mood disorders [44]. Feelings of stigmatization not only result in negative changes in self-image, flaws of logical thinking, social functioning problems, and learned helplessness but also in mood disorders and formation of a depressive triad [41]. Biopsychosocial models explain the relationship between depressive symptoms and the feelings of stigmatization in psoriasis patients. According to this model, both these variables flame each other [38]. Patients who already have some depressive symptoms or self-stigmatization tendency are more likely to suffer from these feelings of psoriasis-related stigmatization [39]. Additionally, stressful life events, positive family history of psoriasis, and a recent infection are risk factors and can initiate the first episode of guttate psoriasis [45].

Any chronic disease could lead to low mood; this could either be due to systemic changes caused by the disease that leads to depression or direct psychological effect of the disease. Depression in psoriasis may be caused by the multiple comorbid diseases associated with it. Bearing the physical aspect of the disease along with the underlying conditions like arthritis, cardiac problems, and other inflammatory conditions may explain why depression occurs in psoriasis. Comorbidities associated with psoriasis include cardiometabolic diseases, gastrointestinal diseases, kidney disease, malignancy, infection, and arthritis [46]. However, their pathogenesis remains to be explored.

In every scenario in life, every individual will react and respond differently. Studies have been carried out to find groups that are more prone to mental and behavioral changes in psoriasis. Women, younger patients, patients with early onset of the disease, and those who self-assess their psoriasis to be severe are especially at risk of mental and behavioral changes in psoriasis [47]. Psoriatic women are more vulnerable to depression with low QoL scores than psoriatic men [45,48]. Regarding men, secretiveness is common; they try to keep their lesions secret by covering them under clothes and avoid the situations where their lesions become visible. They try to hide the truth of their disease from their friends and family; this behavior of trying to hide their disease from family and friends is due to their fear of being rejected by society [39]. In contrast, some studies do not find any significant gender differences regarding stigmatization [8].

A study compared the clinical diagnosis of depression, anxiety, and suicidality among 146,042 mild psoriasis, 3,956 severe psoriasis, and 766,950 control patients. Table 1 shows the results of attributable risks of depression, anxiety, and suicidality per 1,000 person-years among them. The risk was similar between mild and severe psoriasis except for depression. Severe psoriasis showed an increased risk of depression than mild psoriasis [49].

**Table 1:** Attributable risk ^1^Adjusted for age and sex

	Mild Psoriasis	Severe Psoriasis	All Psoriasis
Depression			
Attributable risk^1^ per 1,000 person-years	11.5	25.5	11.8
Anxiety			
Attributable risk^1^ per 1,000 person-years	8.0	8.1	8.1
Suicidality			
Attributable risk^1^ per 1,000 person-years	0.4	0.4	0.4

Psoriasis patients isolate themselves as a result of social reactions from their surroundings due to physical appearance of the disease. Patients try to hide their lesions under the clothes, and avoid going out and seeing people as this would increase the chance of the lesions been seen by others. Those patients going through negative physical and emotional changes due to the disease get depressed, and their self-confidence shatters when they feel that society is not accepting them. Loneliness, accompanied by loss of social support, causes depression. Women are more vulnerable to the psychological aspects of the disease than men. Different comorbid conditions associated with psoriasis may also exacerbate the process of depression in psoriasis. Zięciak et al. discussed the correlation between the feelings of stigmatization in psoriasis patients and their depressive symptoms; they considered factors like gender and skin lesion visibility in their study [39]. Their study consisted of 54 adult men and women suffering from psoriasis who were asked to fill a Feeling of Stigmatization Questionnaire and the Beck Depression Inventory along with marking of the location of their psoriatic lesion on a diagram. On the other hand, Golpour et al. carried a hospital-based case-control study where 100 psoriasis and 100 healthy dermatological controls were studied to investigate the depression and anxiety disorders between them [45]. Spielberger State-Trait Anxiety Scale I-II and Beck Depression Inventory were delivered to both groups. Patients with psoriasis showed higher depression and anxiety scores than the control group.

Limitations

Literature included in this article is limited to humans and the English language. Most of the studies used in the article had small sample sizes. Data collected have emphasized the bidirectional relationship of depression and psoriasis, and there is limited evidence regarding the measures that could be taken to minimize the risk of depression in psoriasis. Not enough randomized control trials have been carried out in this field, and so the literature mainly consisted of the review articles.

## Conclusions

Substantial evidence is present that links psoriasis with depression. This article further explores the relationship between psoriasis and depression. From the discussion so far, it is concluded that psoriasis and depression share multiple common mechanisms and that the two do co-exist. Increased severity of psoriasis can lead to increased depression and vice versa. Females, children, and elderly with psoriasis are more vulnerable to depression than men. Social stigmatization and low self-esteem secondary to psoriasis play a significant role in causing depression in patients with psoriasis.

Studying the relationship between psoriasis and depression holds psychological importance. It can help identify the risk factors that can lead to depression in psoriasis and prepare the dermatologists in advance to deal with them by offering psychological support in conjunction with the treatment of psoriasis, hence minimizing the chances of psychological distress in the patients. Studies regarding the group at risk are controversial, and more research is needed to identify the group so that they can be dealt with special care. Studies regarding the psychological and social support during psoriasis are recommended; this may help the patients to cope with depression and stress during psoriasis.
